# A comprehensive review of immune checkpoint inhibitor-related diabetes mellitus: incidence, clinical features, management, and prognosis

**DOI:** 10.3389/fimmu.2024.1448728

**Published:** 2024-11-04

**Authors:** Lin Zhou, Shuhui Yang, Youtao Li, Cheng Xue, Renping Wan

**Affiliations:** ^1^ Department of Thoracic Surgery, YueBei People’s Hospital, Shaoguan, China; ^2^ Department of Pathology, YueBei People’s Hospital, Shaoguan, China; ^3^ Division of Nephrology, Shanghai Changzheng Hospital, Second Military Medical University (Naval Medical University), Shanghai, China

**Keywords:** immune checkpoint inhibitor, immune-related adverse events, diabetes mellitus, islet autoantibody, diabetic ketoacidosis, hyperglycemia

## Abstract

Immune checkpoint inhibitor-related diabetes mellitus (ICI-DM) is a rare complication that medical oncologists seldom encounter in routine practice. The sporadic nature and intrinsic complexity of ICI-DM make it challenging to analyze comprehensively in experimental settings. In this review, we examine phase 3 clinical trials on ICIs and published case reports of ICI-DM, aiming to summarize its incidence, clinical features, management, and prognosis. Phase 3 clinical trials reveal that the incidence of ICI-DM is higher with combination therapies, such as anti-PD-1 and anti-CTLA-4 or anti-PD-L1, compared to anti-PD-1 monotherapy. ICI-DM typically presents as severe hyperglycemia with a fulminant onset and is often associated with diabetic ketoacidosis, accompanied by unexpectedly low HbA1c and C-peptide levels. ICI-DM shares similarities with classic type 1 diabetes, particularly in terms of autoimmunity and genetic predisposition. This includes a high prevalence of islet autoantibodies and susceptibility to certain HLA haplotypes, often with concurrent endocrine gland dysfunction. This suggests that genetic susceptibility and exposure to ICIs may both be necessary for triggering islet autoimmunity and inducing ICI-DM. Notably, patients with positive islet autoantibodies, such as glutamic acid decarboxylase antibody and islet-associated antigen 2 antibody, tend to experience rapid onset of ICI-DM after ICI exposure. Although patients with ICI-DM generally show a high objective response rate to immunotherapy, a significant proportion also face the need to permanently discontinued treatment. Further research is urgently needed to determine whether permanent discontinuation of immunotherapy is necessary and whether this discontinuation negatively impacts overall survival.

## Introduction

Immune checkpoint inhibitors (ICIs) are widely used for cancer treatment and have marked improved survival of patients with various advanced cancers. The key ICIs in current use consist of monoclonal antibodies targeted to cytotoxic T lymphocyte antigen 4 (anti-CTLA4) and programmed cell death 1/ligand 1 (anti-PD-1/PD-L1), which is a key signaling molecule in immune escape pathways of tumor cells. Owing to dramatically improved survival compared with traditional standard chemotherapy, dozens of anti-CTLA4, anti-PD-1 and anti-PD-L1 antibodies have been approved for several tumor therapies, including non-small cell lung cancer (NSCLC), colorectal cancer, melanoma, etc. However, they can also lead to drug-induced autoimmunity, termed immune-related adverse events (irAEs), which can target virtually any organ system within the body and range in severity from mild to life-threatening. The incidence of potential life-threatening irAEs (grade ≥3) is approximately 10-20% (1, 2).

Endocrinopathies are among the most frequent irAEs. They originate in the immune injury of the endocrine gland and emerge clinically when hormone deficiency reaches a crucial threshold. ICI-DM is one such serious life-altering and life-threatening irAEs. It was first described in 2015 in a case series with anti-PD-1 antibody exposure (3). Subsequently, reports of the clinical syndrome were increasing due to the widespread use of ICIs. ICI-DM and cancer together represent a unique state characterized by nutritional, metabolic, and immunological adjustments, which can affect the treatment of the two diseases. Many problems, for example, what is the criteria for diagnosis and grade of ICI-DM, and whether immunotherapy should be stopped, whether and when glucocorticoids should be used, are controversial. There were conflicting conclusions on the prognosis of patients with ICI-DM. Previous studies have reported that DM is independently a worse prognostic factor for patients with cancers, including liver, endometrial, breast, colorectal, and pancreatic cancer (4–6). However, the development of irAEs is sometimes associated with better ICI treatment outcomes, including ICI-DM (7–10). Although there have been a few retrospective studies on the incidence, clinical features, management, and prognosis of ICI-DM, the small sample size of positive patients on account of the sporadic occurrences of this disease limited the reliability of the conclusions. Herein, we summarize the incidence, clinical features, management, and prognosis of patients with ICI-DM mainly based on published phase 3 clinical trials and published case reports.

## Definition or diagnostic criteria of ICI-DM

ICI-DM includes a new-onset ICI-related T1DM and worsening of prediabetes or T2DM results from ICI exposure. National Comprehensive Cancer Network (NCCN), American Society of Clinical Oncology (ASCO), and European Society for Medical Oncology (ESMO) clinical practice guidelines for the management of toxicities from immunotherapy have only defined and graded ICI-related hyperglycemia not ICI-DM (11, 12). NCCN guidelines proposed measurement of autoantibodies and C-peptide to evaluate and classify ICI-DM if new-onset fasting glucose > 200mg/dL or random blood glucose > 250mg/dL or history of T2DM with fasting/random blood glucose > 250mg/dL. Almost all patients reported with ICI-DM were presented exclusively with rapidly elevated blood glucose due to the rapid destruction of β cells. Therefore, fulfillment of the clinical definition and low or even absence of C-peptide in fasting and postprandial phases can be used as the diagnostic criteria for ICI-DM.

In Japan, ICI-DM was diagnosed based on the criteria of acute-onset T1DM (AT1DM) and fulminant T1DM (FT1DM) set by the Committee of the Japan Diabetes Society (JDS) (13). The main criteria for definite diagnosis of AT1DM and FT1DM are as follows: 1) Occurrence of diabetic ketosis or ketoacidosis soon (approximately 7 days) after the onset of hyperglycemic symptoms. 2) Plasma glucose level ≥16.0 mmol/L (≥288 mg/dL) and HbA1c <8.7% at first visit. 3) Urinary C-peptide excretion <10 μg/day or fasting serum C-peptide level <0.3 ng/mL and <0.5 ng/mL after intravenous glucagon (or after meal) load at onset. 4) Islet-related autoantibodies, such as glutamic acid decarboxylase antibody (anti-GAD), islet-associated antigen 2 antibody (anti-IA2), zinc transporter 8 antibody (anti-ZnT8), anti-islet cell antibody (anti-IC), and insulin autoantibody (anti-I), and the HLA class II haplotypes are valuable for a definite diagnosis.

In China, Shen et al. proposed diagnostic criteria for ICI-DM was that fasting glucose level ≥ 16 mmol/L and HbA1c level < 8.5% at first diagnosis for patients without diabetes history (14). Kotwal et al. proposed to identify cases concerning ICI-DM based on the following criteria (15): (1) New diagnosis of fulminant insulin-dependent diabetes or hyperglycemic crisis. (2) Worsening of prediabetes or T2DM without another attributable reason, defined as an increase in HbA1c value by 10% in 6 months, clinical need for a second antihyperglycemic agent or insulin, DKA, or new-onset ketonuria or ketonemia.

Most of the ICI-DM cases reported in published literature were classified into AT1DM or FT1DM based on the clinical course. Patients with prediabetes or T2DM diagnosed as ICI-DM according to a certain cut-off value of HbA1c and blood glucose was controversial. These specific values were derived from clinical trial protocols and were not necessarily based on evidence. As knowledge about this distinct aspect of ICI-DM accumulates, this definition or diagnostic criteria can be modified.

## Epidemiology

We searched the electronic databases MEDLINE and Pubmed Central to collect all relevant case reports. **Table 1** summarizes the main reported cases of ICI-DM reported in the literature. A total of 109 patients with a diagnosis of ICI-DM were reported when we launched this study, among whom 74 (67.9%) were male, only 11 (10.1%) with a history of DM, 58 (53.2%) cases were induced by anti-PD-1 inhibitors, 31 (28.4%) by anti-PD-1 combined with anti-CTLA4, 10 (9.2%) by anti-PD-L1 and 10 (9.2%) by anti-PD-L1 combined with anti-CTLA4. Different from ordinary T1DM, which increases during childhood and peaks between age 10 years and 14 years (16), ICI-DM is more common in the elderly based on 109 case reports, with a median age of 62 years (range, 53.5-71.5 years). ICI-DM occurred as early as one week or at the latest up to 26 months after ICI initiation (3, 17), with a median time of 13 weeks (range, 6.0-26.3 weeks).

**Table 1:** Clinical characteristics of published cases of patients with ICI-DM.

Characteristic	Value
Number of patients	109
Gender, n (%)	
Male	74 (67.9)
Female	35 (32.1)
Age, (Years)	62 (53.5-71.5)
ICI agents, n (%)	
Anti-PD-1	58 (53.2)
Anti-PD-L1	10 (9.2)
Anti-PD-1+Anti-CTLA4	31 (28.4)
Anti-PD-L1+Anti-CTLA4	10 (9.2)
History of DM, n (%)	11 (10.1)
Onset time of ICI-DM, (Weeks)	13 (6.0-26.3)
DKA, n (%)	
Yes	82 (89.1)
No	10 (10.9)
N/A	17
HbA1c level, %	7.84 (7.10-8.70)
Autoantibody, Positive/Negative	
Anti-GAD	37/57
Anti-IA2	14/51
Anti-ZnT8	2/22
Anti-I	8/40
Anti-IC	5/26
Susceptible HLA haplotype, n (%)	33/7
Yes	33 (82.5)
No	7 (17.5)
N/A	69
Concurrent irAE, n (%)	
Hypothyroidism	25 (22.9)
Hyperthyroidism	2 (1.8)
Hypophysitis	9 (8.3)
Primary adrenal insufficiency	15 (13.8)
Non-endocrine irAE	10 (9.2)
Discontinued immunotherapy, n (%)	
Yes	45 (60.0)
No	30 (40.0)
N/A	34
Tumor response, n (%)	
CR	11 (15.7)
PR	39 (55.7)
SD	10 (14.3)
PD	10 (14.3)
N/A	39

N/A, not available.

The incidence rates of ICI-DM vary with the ICIs used. To estimate the prevalence of ICI-DM, we reviewed phase 3 clinical trials including patients previously untreated with ICI. Those trials, in which immune-mediated adverse events had not been completely reported or the follow-up time was less than two years, were excluded. The type of cancer, with or without chemotherapy, and regimens of chemotherapy were not questioned. In phase 3 clinical trials described in **Table 2**, the incidence rate of ICI-DM induced by Ipilimumab, the most widely used anti-CTLA4 antibody, was extremely low. We reviewed eight phase 3 clinical trials involving 3701 patients treated with Ipilimumab with or without chemotherapy, and only one (0.027%) patient was identified as ICI-DM in CheckMate-238 trial (18–25). Anti-PD-1 antibodies were more likely to induce ICI-DM compared to anti-CTLA4 inhibitors. In sixteen phase 3 clinical trials involving 5520 patients treated with Nivolumab, a total of 26 (0.47%) patients were diagnosed with ICI-DM. Among 11403 patients treated with Pembrolizumab in twenty-eight phase 3 clinical trials, 58 (0.51%) were confirmed to have ICI-DM. Among sixteen phase 3 clinical trials included 6538 patients who received Atezolizumab, the most widely used anti-PD-L1 antibody, 52 (0.80%) patients were identified as ICI-DM. It seems that anti-PD-L1 antibodies induced a statistically higher prevalence of ICI-DM compared with anti-PD-1(*P*=0.007). However, analysis of 9 phase 3 clinical trials comprising 3447 eligible patients to assess the efficacy and safety of durvalumab, another selective, high-affinity PD-L1 inhibitor, revealed that a total of 9 (0.26%) patients were diagnosed as ICI-DM, which was statistically lower than that induced by Atezolizumab (P=0.001). Despite the combination of anti-PD-1 and anti-CTLA4 inhibitors being more effective than either agent alone, dual immune checkpoint blockade therapy induces a higher prevalence of ICI-DM. We reviewed twelve phase 3 clinical trials on dual ICI consisting of Nivolumab or Pembrolizumab plus Ipilimumab, and the prevalence of ICI-DM was close to 1% (51/5116). The highest incidence rate of ICI-DM was up to 5.7% (6/106) in Checkmate-920, however, the small sample size indicated that it was not universal. A trend for increased incidence of ICI-DM (0.44%, 11/2518) with dual ICI combination consisting of durvalumab and tremelimumab, an anti-CTLA4 antibody, did not reach statistical significance compared with durvalumab monotherapy (*P*=0.246). However, the small sample size of clinical trials evaluated durvalumab with or without tremelimumab and the short median treatment duration (<6 months) in these clinical trials limit the representativeness of the results.

**Table 2:** The incidence rates of ICI-DM in phase 3 clinical trials including patients previously untreated with ICI.

	Clinic trials	Malignancy	ICI-DM		Number of participants.
			All grades	Grades ≥3	
Nivolumab	Checkmate-017	Lung squamous cell cancer	0	0	131
	Checkmate-057	Nonsquamous non-small cell lung cancer	0	0	287
	Checkmate-066	Melanoma	1	0	206
	Checkmate-067	Melanoma	1	1	313
	Checkmate-078	Non-small cell lung cancer	1	0	337
	Checkmate-141	Squamous-cell carcinoma of the head and neck	0	0	236
	Checkmate-227	Non-small cell lung cancer	3	3	562
	Checkmate-238	Melanoma	2	1	452
	Checkmate-459	Hepatocellular carcinoma	0	0	367
	Checkmate-915	Melanoma	11	9	917
	Checkmate-9ER	Renal-cell carcinoma	0	0	320
	Confirm	Malignant mesothelioma	0	0	221
	Attraction-2	Gastric or gastro-oesophageal junction cancer	2	2	330
	Attraction-3	Oesophageal squamous cell carcinoma	1	1	209
	Attraction-4	Gastric or gastro-oesophageal junction cancer	0	0	359
	Tasuki-52	Non-small cell lung cancer	4	3	273
Total			26 (0.47%)	20	5520
Pembrolizumab	Keynote-006	Melanoma	2	2	555
	Keynote-010	Non-small cell lung cancer	3	3	682
	Keynote-024	Non-small cell lung cancer	1	1	154
	Keynote-040	Squamous-cell carcinoma of the head and neck	0	0	246
	Keynote-042	Non-small cell lung cancer	0	0	636
	Keynote-045	Urothelial carcinoma	1	1	266
	Keynote-052	Urothelial carcinoma	4	4	370
	Keynote-054	Melanoma	5	5	509
	Keynote-061	Gastric or gastro-oesophageal junction cancer	1	0	294
	Keynote-062	Urothelial carcinoma	3	N/A	504
	Keynote-091	Non-small cell lung cancer	1	N/A	580
	Keynote-119	Breast cancer	1	1	309
	Keynote-181	Esophageal Cancer	1	N/A	314
	Keynote-189	Nonsquamous non-small cell lung cancer	2	2	405
	Keynote-240	Hepatocellular carcinoma	1	1	279
	Keynote-252	Melanoma	3	2	705
	Keynote-394	Hepatocellular carcinoma	0	0	299
	Keynote-407	Lung squamous-cell cancer	0	0	169
	Keynote-426	Renal-cell carcinoma	1	1	429
	Keynote-564	Renal-cell carcinoma	9	9	488
	Keynote-590	Esophageal Cancer	1	1	370
	Keynote-598	Non-small cell lung cancer	0	0	281
	Keynote-604	Small cell lung cancer	1	1	223
	Keynote-716	Melanoma	2	2	483
	Keynote-775	Endometrial cancer	4	N/A	506
	Keynote-826	Cervical Cancer	2	2	307
	Clear	Renal-cell carcinoma	2	1	352
	Mastkey-265	Melanoma	7	5	688
Total			58 (0.51%)	44	11403
Atezolizumab	Impower-010	Non-small cell lung cancer	4	0	495
	Impower-110	Non-small cell lung cancer	5	2	286
	Impower-130	Nonsquamous non-small cell lung cancer	6	3	473
	Impower-131	Lung squamous-cell cancer	7	4	666
	Impower-132	Nonsquamous non-small cell lung cancer	3	2	291
	Impower-133	Small cell lung cancer	1	0	198
	Impower-150	Nonsquamous non-small cell lung cancer	1	0	393
	Engot-OV29	Ovarian cancer	2	N/A	408
	Imagyn050	Ovarian cancer	2	1	642
	Impassion-130	Breast cancer	1	1	452
	Impassion-131	Breast cancer	5	4	432
	Immotion-010	Renal-cell carcinoma	4	3	390
	Imvigor-010	Urothelial carcinoma	1	1	390
	Imvigor211	Urothelial carcinoma	0	0	459
	Imbrave-150	Hepatocellular carcinoma	8	1	329
	BFAST	Non-small cell lung cancer	2	0	234
Total			52 (0.8%)	22	6538
Durvalumab	DANUBE	Urothelial carcinoma	2	0	345
	CASPIAN	Small-cell lung cancer	4	4	265
	KESTREL	Squamous-cell carcinoma of the head and neck	0	0	413
	HIMALAYA	Hepatocellular carcinoma	0	0	388
	MYSTIC	Non-small cell lung cancer	0	0	369
	POSEIDON	Non-small cell lung cancer	1	1	334
	CALLA	cervical cancer	0	0	385
	DUO-E	Endometrial Cancer	1	N/A	473
	PACIFIC	Non-small cell lung cancer	1	N/A	475
Total			9 (0.26%)	5	3447
Nivolumab or Pembrolizumab + Ipilimumab	Checkmate-067	Melanoma	3	2	313
	Checkmate-214	Renal-cell carcinoma	6	2	547
	Checkmate-227	Non-small cell lung cancer	5	5	576
	Checkmate-401	Melanoma	3	2	533
	Checkmate-651	Squamous-cell carcinoma of the head and neck	4	4	468
	Checkmate-743	Malignant mesothelioma	1	1	303
	Checkmate-817	Non-small cell lung cancer	4	4	589
	Checkmate-914	Renal-cell carcinoma	9	8	404
	Checkmate-915	Melanoma	8	6	916
	Checkmate-920	Renal-cell carcinoma	6	4	106
	Checkmate-9LA	Non-small cell lung cancer	0	0	361
	Keynote-598	Non-small cell lung cancer	2	2	282
Total			51 (1.0%)	40	5116
Durvalumab+Tremelimumab	DANUBE	Urothelial carcinoma	1	1	340
	CASPIAN	Small-cell lung cancer	2	2	266
	KESTREL	Squamous-cell carcinoma of the head and neck	2	N/A	413
	HIMALAYA	Hepatocellular carcinoma	0	0	388
	MYSTIC	Non-small cell lung cancer	2	1	371
	POSEIDON	Non-small cell lung cancer	1	1	330
	NEPTUNE	Non-small cell lung cancer	3	3	410
Total			11(0.44%)	8	2518

N/A, not available.

The prevalence of ICI-DM in most retrospective studies was also lower than 1%, which was consistent with the prevalence observed in clinical trials. For instance, several large sample single-center retrospective studies estimated the prevalence of ICI-DM to be 0.25%, 0.48%, and 0.9%, respectively (15, 26–28).

Multiple studies have described the effect of geographical location, race, and ethnicity on the incidence rate of classic T1DM. A higher than 360-fold difference was noted among the 100 countries ranging from a low of 0.1/100,000 per year in China and Venezuela to a high of 36.5/100,000 in Finland and Sardinia (29). Boswell L et al. also reported that non-Asian ethnicity was predominant in ICI-related diabetes patients (17). In phase 3 clinical trials with mostly Chinese patients, such as Orient-11, 31, 32 and Rationale-306 et al., no case of ICI-DM have been reported, which appeared to reflect a different degree of genetic susceptibility due to ethnic origin (30–33). Furthermore, there was also no case of ICI-DM had been recorded in subgroup of Japanese patients in Impassion-031 and Impower-010 (34, 35). But this hypothesis that ethnic origin is one of contributory cause of diversity in terms of the prevalence of ICI-DM lack direct evidence based on large sample survey.

## Islet autoimmunity

ICI-DM is essentially a form of autoimmune diabetes mellitus, characterized by the development of an immune response against specific β cell antigens. Several islet autoantibodies have been described to be associated with ICI-DM, and include anti-GAD, anti-ZnT8, anti-IA2, ani-I, and anti-IC. Anti-GAD is especially important. This autoantibody has the highest prevalence in patients with positive islet autoantibodies, and it may be associated with the rapid onset of ICI-DM (28).

**Figure 1:**
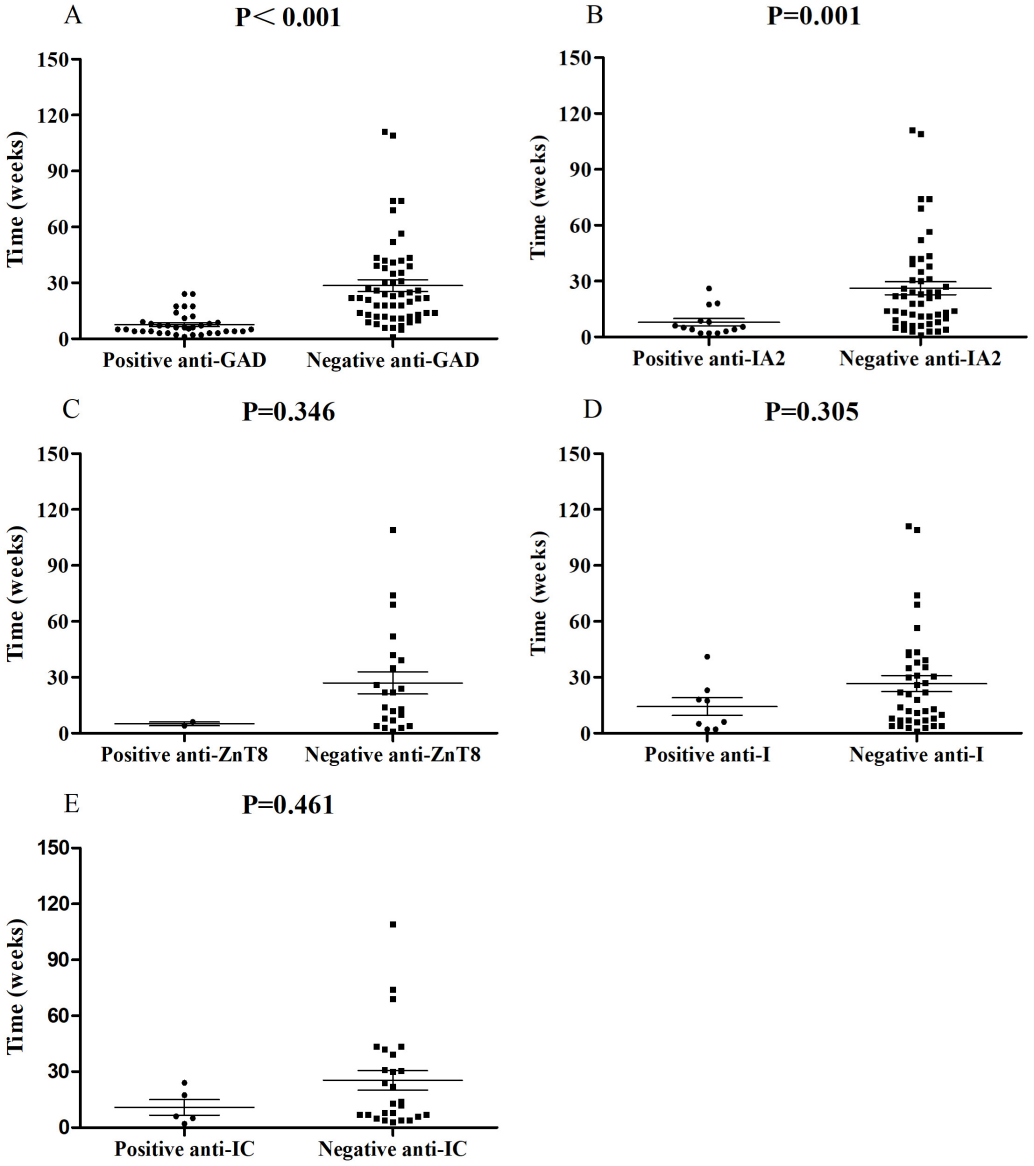
Comparisons of onset time of ICI-DM since ICI exposure between patients with positive anti-GAD **(A)**, anti-IA2 **(B)**, anti-ZnT8 **(C)**, anti-I **(D)** and anti-IC **(E)** autoantibodies and negative controls.

In this study, we investigated possible associations between autoantibody status and clinical features through published case reports. Anti-GAD (39.4%, 37/94) was the most common autoantibody followed by anti-IA2 (21.5%, 14/65), anti-I (16.7%, 8/48), anti-ZnT8 (8.3%, 2/24) and anti-IC (16.1%, 5/31), complying with prior studies (28). Anti-IA2, anti-I, anti-ZnT8 and anti-IC were commonly accompanied by positive anti-GAD. Univariate analysis showed that, compared with negative anti-GAD patients, patients with positive anti-GAD exhibited a shorter period from the start of ICI treatment to a diagnosis of ICI-DM (median time, 6.0 vs 22 weeks, *P*<0.001) (**Figure 1A**). Moreover, 73.0% (27/37) of patients with positive anti-GAD have developed ICI-DM within 2 months after the initiation of ICI treatment. A patient with a high level of anti-GAD was even admitted to the emergency department for ketoacidosis within a week after the first infusion of Nivolumab (3). Strikingly, several case reports have shown that anti-GAD was positive before the initiation of treatment with ICIs (36, 37). Therefore, islet autoantibody testing before the start of ICI treatment may contribute to screening patients at high risk for ICI-DM. The univariate analysis also indicated that positive anti-IA2 (*P*=0.001) (**Figure 1B**) experienced a more rapid onset of disease compared with negative cases, but this phenomenon was not seen in patients with positive anti-ZnT8 (*P*=0.346) (**Figure 1C**), anti-I (*P*=0.305) (**Figure 1D**) and anti-IC (*P*=0.461) (**Figure 1E**). Due to most patients did not complete all the five autoantibodies testing, we were unable to perform further multivariable analysis.

## Genetic factors

Susceptibility to ICI-DM has a strong genetic component, with the *HLA* class II haplotypes accounting for up to 50% of the disease risk (28). The *DR3-DQ2* and *DR4-DQ8* haplotypes have been described as the major risk factor for classicT1DM as well as ICI-DM, while the *DR4-DQ4* and *DR9-DQ9* haplotypes are linked with fulminant diabetes in Asians (38, 39). Many of these genes are associated with other autoimmune diseases such as autoimmune thyroiditis and rheumatoid arthritis, which co-occur with T1DM at rates greater than would be expected by chance (16).

**Figure 2:**
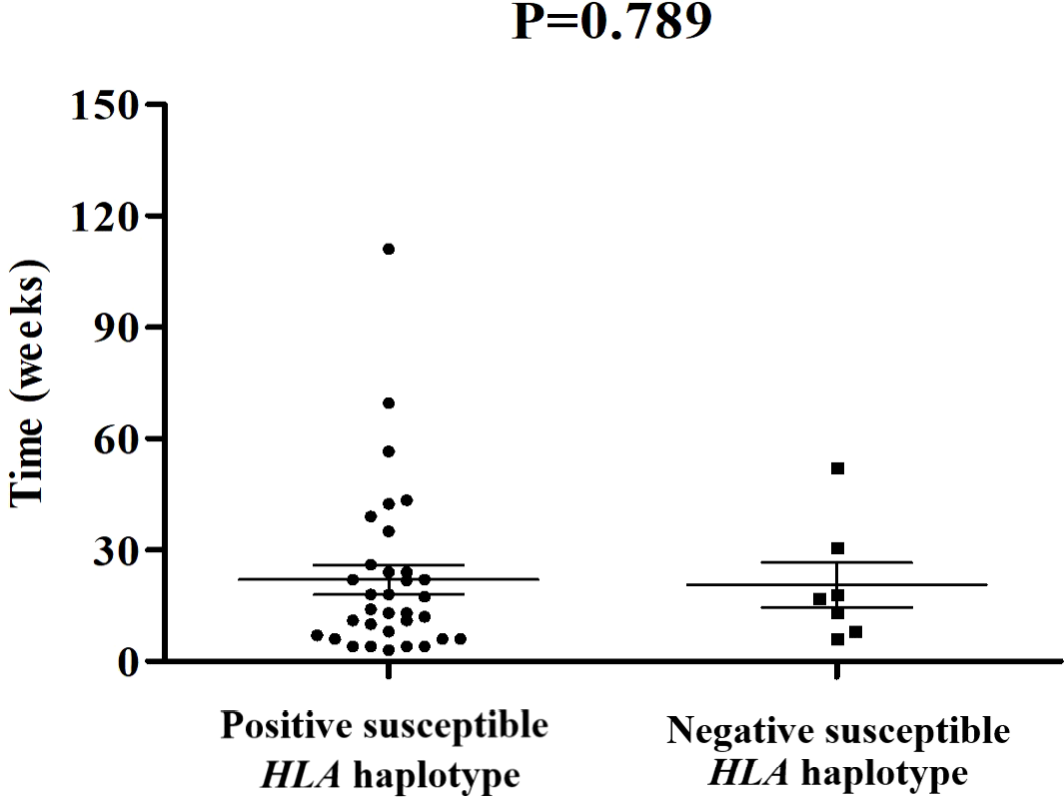
Comparisons of onset time of ICI-DM since ICI exposure between patients with susceptible HLA haplotype and negative controls.

Among these 109 patients with ICI-DM, *HLA*-typing analyses were performed in 40 patients. Predisposing *HLA* haplotype occurred in 82.5% (33/40) of cases. The median period from initiation of ICI treatment to a diagnosis of ICI-DM in patients with susceptible HLA haplotype was 14 versus 17 weeks in patients without susceptible *HLA* haplotype (*P*=0.789) (**Figure 2**). Moreover, there was no significant statistical difference in the prevalence of positive anti-GAD between the two groups (38.7% vs. 57.1%, *P*=0.425). Remarkably, a series of case reports showed that at least 51.5% (17/33) of patients with susceptible *HLA* haplotype experienced concurrent endocrine irAEs, which was similar to classic T1DM. This performance is consistent with the theory from Eisenbarth et al. that genetic susceptibility and exposure to environmental triggers islet autoimmunity are necessary conditions for inducing autoimmune DM (40). Although screening with susceptible *HLA* haplotype before ICI initiation is currently not recommended, *HLA*-typing may contribute to raising awareness among the physicians administering ICI.

## Clinical features

The most common clinical manifestations in patients with ICI-DM are fatigue, polyuria, polydipsia, hypotension, and hypersomnia, and the most severe forms connect closely with ketoacidosis. Symptoms of DKA may include excessive thirst, frequent urination, general weakness, vomiting, confusion, abdominal pain, dry skin, dry mouth, increased heart rate, and fruity odor on the breath. Two recent reviews indicated that the incidence of ICIs related to DKA was 57% and 76% (41, 42). According to this series of case reports of ICI-DM, a total of 93 cases had been reported information of blood pH, among whom 66 patients (71%) were diagnosed with DKA by authors or eligible for DKA according to guidelines from the Joint British Diabetes Society for Inpatient Care (43).

HbA1c and C-peptide, measurement of average blood glucose over the past 8 to 12 weeks, and endogenous insulin production respectively, are important diagnosis proof of ICI-DM. Owing to the rapid β cell dysfunction, HbA1c, and C-peptide are generally inappropriately low for the degree of hyperglycemia at the time of diagnosis. A total of 79 cases had been reported with the detection result of HbA1c, among which 7 patients (8.9%) were at the normal range. Elevated HbA1c was detected in 72 patients (91.1%), with a median value of 7.84%. There were only 9 patients (11.4%) who had a concentration of HbA1c more than 10%. In this study, the majority of patients with ICI-DM have been recorded with an extremely low or even undetectable C-peptide level. Some authors have proposed testing of C-peptide level for aiding diagnosis and repeating for confirming if the β-cell function could be recovered (44, 45).

## Management

Due to the potential to lead to life-threatening consequences, patients who experience severe hyperglycemia or DKA on ICIs should be hospitalized. As impaired β-cell function is generally irreversible, insulin replacement should be started as soon as possible and typically required lifelong. DKA requires continuous intravenous insulin injections rather than subcutaneous injections. Moreover, management of DKA also includes IV fluid with or without potassium supplementation, and hourly testing of glucose, serum ketones, blood pH, and electrolyte. After the improvement of DKA, daily subcutaneous insulin injections instead of intravenous injections can be started refer to guidelines for the management of T1DM or consult with an endocrinologist.

Although severe irAEs in other endocrine organs are often treated with high doses of glucocorticoids, this agent is not recommended for managing ICI-DM because there is no evidence that the use could improve the islet function or the survival outcomes (46, 47). Management of ICI-DM is complicated by concurrent severe irAEs, especially additional endocrine-related AEs, and the administration of medications. Based on data in these case reports, other severe irAEs were observed in at least 50.5% (55/109) of patients, 60% (33/55) of whom were endocrine-related. Concurrent severe endocrine irAEs, most commonly hypothyroidism, followed by adrenal insufficiency, hypophysitis and hyperthyroidism. Eleven patients were diagnosed with dysfunction of at least three endocrine organs. Clinicians should be aware that additional endocrine irAEs present with high frequency in individuals with ICI-DM, and screen for thyroid, pituitary and adrenal gland disease. According to prior studies, improvement of the pituitary-thyroid and pituitary-gonadal axis was observed in up to 50-60% of patients, and recovery of the pituitary-adrenal axis occurred in a few cases (48–50). Thus, sometimes, high doses of glucocorticoids for concurrent AEs are needed. It may help to mitigate symptoms of acute inflammation in the setting of hypophysitis, adrenalitis, or in some cases, thyrotoxicosis. If patients need glucocorticoids for the treatment of concurrent AEs, blood glucose levels should be monitored more carefully. Due to this potential complexity, the close collaboration between oncologists and endocrinologists plays an important role in the management of these severe or complex cases.

Upon treatment of patients with insulin, symptoms of DKA may be expected to improve or resolve in 1 week. Most of the Guidelines recommended that the use of ICIs in patients with ICI-DM can be restarted combined with insulin replacement therapy once the general conditions are stabilized by treatment (51, 52). However, no randomized controlled trials have assessed the long-term toxicity and effectiveness of such a combination regimen. Among 75 patients with data on follow-up therapy in these case reports, 45 (60.0%) patients permanently discontinued ICI therapy, but only seven of them were accompanied by concurrent severe non-endocrine irAEs, such as immune-related encephalitis, hepatitis, pneumonia, and colonitis. Symptom severity stratification according to the Common Terminology Criteria for Adverse Events (CTCAE) may be responsible for this. For example, a total of 207 patients in 78 phase 3 clinical trials were diagnosed with ICI-DM, among whom 139 (67.1%) were defined as grade ≥3 irAE, then ICIs should be discontinued according to general guidelines for the management of irAEs. The clinical consequences arising from the resulting insulin deficiency are usually permanent, therefore, ICI-DM does not fit into a five-tier symptom severity stratification diagnostically and therapeutically based on CTCAE (53). Because β cell failure can be managed with exogenous insulin replacement, clinicians need to balance the benefits of continued ICI therapy, such as the possibility of shrinking the tumor or survival improvement, with risks, such as deteriorating concurrent irAEs.

## Outcomes

In 78 phase 3 clinical trials included in this study, although 67.1% (139/207) were regarded as serious irAEs of grade ≥3, there were no ICI-DM-related death events. Similarly, among 109 patients in case reports, although 27 were referred to the emergency department or the Intensive Care Unit after the onset of ICI-DM, there were no patients who died from DKA. It indicated that early diagnosis and prompt management could be crucial for improved treatment outcomes of ICI-DM.

Whether developing ICI-DM is linked to treatment response and survival outcomes is ambiguous. Previous studies have reported that DM is independently a worse prognostic factor for patients with cancers and infectious diseases. Nevertheless, some studies showed that certain irAEs might be positive prognostic factors of treatment response and overall survival (7, 8, 54, 55). By reviewing data from 109 patients in case reports, assessment for tumor response was acquired in 70 cases, among whom 11 (15.7%) with a complete response (CR), 39 (55.7%) with a partial response (PR), 10 (14.3%) with a stable disease (SD), 10 (14.3%) with progressive disease (PD). Although these case series included different kinds of cancer, 71.4% (50/70) of patients with ICI-DM achieved an objective response, which was significantly higher than that of any phase 3 clinical trial involving immunotherapy. This result supports the view that irAEs were a biomarker of treatment response, complying with prior studies.

## Conclusions

In conclusion, ICI-DM is a rare complication that a medical oncologist rarely sees in his routine practice. Fulminant severe hyperglycemia, DKA and inappropriately low HbA1c and C-peptide can be the main clinical clue. Rapid diagnosis followed by insulin replacement should improve a patient’s outcome. ICI-DM resembles classic T1DM in terms of autoimmunity and genetic component, reflecting in the presence of a high rate of patients with positive islet autoantibodies, susceptible HLA haplotype, and concurrent other endocrine gland damage. The improved understanding of the mechanisms of ICI action interacting with islet autoimmunity and genetic susceptibility should contribute to the recognition of the etiology and pathogenesis of ICI-DM and classic T1DM. Patients with ICI-DM presented with a high objective response rate of tumor response to ICI treatment, however, the proportion of patients permanently discontinued immunotherapy was also high. Further research is urgently needed to identify whether permanently discontinued immunotherapy is required and whether ICI discontinuance deteriorates the OS of the patient.

## References

[1] Naidoo J Page D Li B Connell L Schindler K Lacouture M. Toxicities of the anti-PD-1 and anti-PD-L1 immune checkpoint antibodies. Ann oncology: Off J Eur Soc Med Oncol. (2015) 26:2375–91. doi: 10.1093/annonc/mdv383 PMC6267867 26371282

[2] Ye W Olsson-Brown A Watson R Cheung V Morgan R Nassiri I. Checkpoint-blocker-induced autoimmunity is associated with favourable outcome in metastatic melanoma and distinct T-cell expression profiles. Br J Cancer. (2021) 124:1661–9. doi: 10.1038/s41416-021-01310-3 PMC8110747 33723392

[3] Hughes J Vudattu N Sznol M Gettinger S Kluger H Lupsa B. Precipitation of autoimmune diabetes with anti-PD-1 immunotherapy. Diabetes Care. (2015) 38:e55–7. doi: 10.2337/dc14-2349 PMC4370325 25805871

[4] Pearson-Stuttard J Papadimitriou N Markozannes G Cividini S Kakourou A Gill D. Type 2 diabetes and cancer: an umbrella review of observational and mendelian randomization studies. Cancer epidemiology Biomarkers prevention: Publ Am Assoc Cancer Research cosponsored by Am Soc Prev Oncol. (2021) 30:1218–28. doi: 10.1158/1055-9965.EPI-20-1245 PMC9398112 33737302

[5] Tsilidis K Kasimis J Lopez D Ntzani E Ioannidis J. Type 2 diabetes and cancer: umbrella review of meta-analyses of observational studies. BMJ (Clinical Res ed). (2015) 350:g7607. doi: 10.1136/bmj.g7607 25555821

[6] Campbell P Newton C Patel A Jacobs E Gapstur S. Diabetes and cause-specific mortality in a prospective cohort of one million U.S. adults. Diabetes Care. (2012) 35:1835–44. doi: 10.2337/dc12-0002 PMC3425000 22699290

[7] Teulings H Limpens J Jansen S Zwinderman A Reitsma J Spuls P. Vitiligo-like depigmentation in patients with stage III-IV melanoma receiving immunotherapy and its association with survival: a systematic review and meta-analysis. J Clin oncology: Off J Am Soc Clin Oncol. (2015) 33:773–81. doi: 10.1200/JCO.2014.57.4756 25605840

[8] Sanlorenzo M Vujic I Daud A Algazi A Gubens M Luna S. Pembrolizumab cutaneous adverse events and their association with disease progression. JAMA Dermatol. (2015) 151:1206–12. doi: 10.1001/jamadermatol.2015.1916 PMC5061067 26222619

[9] Lo J Fisher D Flaherty K. Prognostic significance of cutaneous adverse events associated with pembrolizumab therapy. JAMA Oncol. (2015) 1:1340–1. doi: 10.1001/jamaoncol.2015.2274 PMC4679487 26270186

[10] Ornelas M Borges-Canha M Gouveia P Ferreira M Resende E Sá M. Immune checkpoint inhibitor-induced endocrinopathies: a possible indicator of improved survival. Arch Endocrinol Metab. (2023) 67:e000654. 37364153 10.20945/2359-3997000000654 PMC10661004

[11] Haanen J Obeid M Spain L Carbonnel F Wang Y Robert C. Management of toxicities from immunotherapy: ESMO Clinical Practice Guideline for diagnosis, treatment and follow-up. Ann oncology: Off J Eur Soc Med Oncol. (2022) 33:1217–38. doi: 10.1016/j.annonc.2022.10.001 36270461

[12] Brahmer J Lacchetti C Schneider B Atkins M Brassil K Caterino J. Management of immune-related adverse events in patients treated with immune checkpoint inhibitor therapy: american society of clinical oncology clinical practice guideline. J Clin oncology: Off J Am Soc Clin Oncol. (2018) 36:1714–68. doi: 10.1200/JCO.2017.77.6385 PMC6481621 29442540

[13] Imagawa A Hanafusa T Awata T Ikegami H Uchigata Y Osawa H. Report of the Committee of the Japan Diabetes Society on the Research of Fulminant and Acute-onset Type 1 Diabetes Mellitus: New diagnostic criteria of fulminant type 1 diabetes mellitus (2012). J Diabetes Invest. (2012) 3:536–9. 10.1111/jdi.12024 PMC4015434 24843620

[14] Shen M Chen D Zhao R Zheng X Gu Y Yang T. Real-world adherence to toxicity management guidelines for immune checkpoint inhibitor-induced diabetes mellitus. Front Endocrinol. (2023) 14:1213225. doi: 10.3389/fendo.2023.1213225 PMC10405819 37554766

[15] Kotwal A Haddox C Block M Kudva Y. Immune checkpoint inhibitors: an emerging cause of insulin-dependent diabetes. BMJ Open Diabetes Res Care. (2019) 7:e000591. doi: 10.1136/bmjdrc-2018-000591 PMC6398813 30899528

[16] Quattrin T Mastrandrea L Walker L. Type 1 diabetes. Lancet (London England). (2023) 401:2149–62. doi: 10.1016/S0140-6736(23)00223-4 37030316

[17] Boswell L Casals G Blanco J Jiménez A Aya F de Hollanda A. Onset of fulminant type 1 diabetes mellitus following hypophysitis after discontinuation of combined immunotherapy. A Case Rep J Diabetes Invest. (2021) 12:2263–6. doi: 10.1111/jdi.v12.12 PMC8668074 34048145

[18] Larkin J Chiarion-Sileni V Gonzalez R Grob J Rutkowski P Lao C. Five-year survival with combined nivolumab and ipilimumab in advanced melanoma. New Engl J Med. (2019) 381:1535–46. doi: 10.1056/NEJMoa1910836 31562797

[19] Eggermont A Chiarion-Sileni V Grob J Dummer R Wolchok J Schmidt H. Adjuvant ipilimumab versus placebo after complete resection of high-risk stage III melanoma (EORTC 18071): a randomised, double-blind, phase 3 trial. Lancet Oncol. (2015) 16:522–30. doi: 10.1016/S1470-2045(15)70122-1 25840693

[20] Ascierto P Del Vecchio M Mackiewicz A Robert C Chiarion-Sileni V Arance A. Overall survival at 5 years of follow-up in a phase III trial comparing ipilimumab 10 mg/kg with 3 mg/kg in patients with advanced melanoma. J immunotherapy Cancer. (2020) 8. doi: 10.1136/jitc-2019-000391 PMC7279645 32503946

[21] Weber J Mandala M Del Vecchio M Gogas H Arance A Cowey C. Adjuvant nivolumab versus ipilimumab in resected stage III or IV melanoma. New Engl J Med. (2017) 377:1824–35. doi: 10.1056/NEJMoa1709030 28891423

[22] Fizazi K Drake C Beer T Kwon E Scher H Gerritsen W. Final analysis of the ipilimumab versus placebo following radiotherapy phase III trial in postdocetaxel metastatic castration-resistant prostate cancer identifies an excess of long-term survivors. Eur Urol. (2020) 78:822–30. doi: 10.1016/j.eururo.2020.07.032 PMC8428575 32811715

[23] Govindan R Szczesna A Ahn M Schneider C Gonzalez Mella P Barlesi F. Phase III trial of ipilimumab combined with paclitaxel and carboplatin in advanced squamous non-small-cell lung cancer. J Clin oncology: Off J Am Soc Clin Oncol. (2017) 35:3449–57. doi: 10.1200/JCO.2016.71.7629 28854067

[24] Eggermont A Chiarion-Sileni V Grob J Dummer R Wolchok J Schmidt H. Prolonged survival in stage III melanoma with ipilimumab adjuvant therapy. New Engl J Med. (2016) 375:1845–55. doi: 10.1056/NEJMoa1611299 PMC5648545 27717298

[25] Reck M Luft A Szczesna A Havel L Kim S Akerley W. Phase III randomized trial of ipilimumab plus etoposide and platinum versus placebo plus etoposide and platinum in extensive-stage small-cell lung cancer. J Clin oncology: Off J Am Soc Clin Oncol. (2016) 34:3740–8. doi: 10.1200/JCO.2016.67.6601 27458307

[26] Marsiglio J McPherson J Kovacsovics-Bankowski J Jeter C Vaklavas U Swami U. A single center case series of immune checkpoint inhibitor-induced type 1 diabetes mellitus, patterns of disease onset and long-term clinical outcome. Front Immunol. (2023) 14:1229823. doi: 10.3389/fimmu.2023.1229823 37671166 PMC10475559

[27] Silverstein J Wright F Wang M Young A Kim D De Dios K. Evaluating survival after hospitalization due to immune-related adverse events from checkpoint inhibitors. oncologist. (2023) 28:e950–e9. doi: 10.1093/oncolo/oyad135 PMC10546826 37335906

[28] Stamatouli A Quandt Z Perdigoto A Clark P Kluger H Weiss S. Collateral damage: insulin-dependent diabetes induced with checkpoint inhibitors. Diabetes. (2018) 67:1471–80. doi: 10.2337/dbi18-0002 PMC6054443 29937434

[29] Ali M Al Suwaidi J. Racial and ethnic differences in cardiovascular disease and outcome in type 1 diabetes patients. Expert Rev Endocrinol Metab. (2019) 14:225–31. doi: 10.1080/17446651.2019.1613887 31081398

[30] Lu S Wu L Jian H Chen Y Wang Q Fang J. Sintilimab plus bevacizumab biosimilar IBI305 and chemotherapy for patients with EGFR-mutated non-squamous non-small-cell lung cancer who progressed on EGFR tyrosine-kinase inhibitor therapy (ORIENT-31): first interim results from a randomised, double-blind, multicentre, phase 3 trial. Lancet Oncol. (2022) 23:1167–79. doi: 10.1016/S1470-2045(22)00382-5 35908558

[31] Ren Z Xu J Bai Y Xu A Cang S Du C. Sintilimab plus a bevacizumab biosimilar (IBI305) versus sorafenib in unresectable hepatocellular carcinoma (ORIENT-32): a randomised, open-label, phase 2-3 study. Lancet Oncol. (2021) 22:977–90. doi: 10.1016/S1470-2045(21)00252-7 34143971

[32] Xu J Kato K Raymond E Hubner R Shu Y Pan Y. Tislelizumab plus chemotherapy versus placebo plus chemotherapy as first-line treatment for advanced or metastatic oesophageal squamous cell carcinoma (RATIONALE-306): a global, randomised, placebo-controlled, phase 3 study. Lancet Oncol. (2023) 24:483–95. doi: 10.1016/S1470-2045(23)00108-0 37080222

[33] Yang Y Wang Z Fang J Yu Q Han B Cang S. Efficacy and Safety of Sintilimab Plus Pemetrexed and Platinum as First-Line Treatment for Locally Advanced or Metastatic Nonsquamous NSCLC: a Randomized, Double-Blind, Phase 3 Study (Oncology pRogram by InnovENT anti-PD-1-11). J Thorac oncology: Off Publ Int Assoc Study Lung Cancer. (2020) 15:1636–46. doi: 10.1016/j.jtho.2020.07.014 32781263

[34] Kenmotsu H Sugawara S Watanabe Y Saito H Okada M Chen-Yoshikawa T. Adjuvant atezolizumab in Japanese patients with resected stage IB-IIIA non-small cell lung cancer (IMpower010). Cancer Sci. (2022) 113:4327–38. doi: 10.1111/cas.v113.12 PMC9746048 36062851

[35] Saji S Ohsumi S Ito M Hayashi N Kobayashi K Masuda N. Subgroup analysis of Japanese patients in a phase III randomized, controlled study of neoadjuvant atezolizumab or placebo, combined with nab-paclitaxel and anthracycline-based chemotherapy in early triple-negative breast cancer (IMpassion031). Japanese J Clin Oncol. (2022) 52:1124–33. doi: 10.1093/jjco/hyac098 PMC9538755 35750038

[36] Godwin J Jaggi S Sirisena I Sharda P Rao A Mehra R. Nivolumab-induced autoimmune diabetes mellitus presenting as diabetic ketoacidosis in a patient with metastatic lung cancer. J immunotherapy Cancer. (2017) 5:40. doi: 10.1186/s40425-017-0245-2 PMC5433051 28515940

[37] Yamaguchi H Miyoshi Y Uehara Y Fujii K Nagata S Obata Y. Case of slowly progressive type 1 diabetes mellitus with drastically reduced insulin secretory capacity after immune checkpoint inhibitor treatment for advanced renal cell carcinoma. Diabetol Int. (2021) 12:234–40. doi: 10.1007/s13340-020-00459-1 PMC7943686 33786278

[38] Steck A Rewers M. Genetics of type 1 diabetes. Clin Chem. (2011) 57:176–85. doi: 10.1373/clinchem.2010.148221 PMC4874193 21205883

[39] Tsutsumi C Imagawa A Ikegami H Makino H Kobayashi T Hanafusa T. Class II HLA genotype in fulminant type 1 diabetes: A nationwide survey with reference to glutamic acid decarboxylase antibodies. J Diabetes Invest. (2012) 3:62–9. doi: 10.1111/j.2040-1124.2011.00139.x PMC4014934 24843547

[40] Eisenbarth G. Type I diabetes mellitus. A chronic autoimmune disease. New Engl J Med. (1986) 314:1360–8. doi: 10.1056/NEJM198605223142106 3517648

[41] Liao D Liu C Chen S Liu F Li W Shangguan D. Recent advances in immune checkpoint inhibitor-induced type 1 diabetes mellitus. Int Immunopharmacol. (2023) 122:110414. doi: 10.1016/j.intimp.2023.110414 37390646

[42] de Filette J Pen J Decoster L Vissers T Bravenboer B Van der Auwera B. Immune checkpoint inhibitors and type 1 diabetes mellitus: a case report and systematic review. Eur J Endocrinol. (2019) 181:363–74. doi: 10.1530/EJE-19-0291 PMC6709545 31330498

[43] Dhatariya K. The management of diabetic ketoacidosis in adults-An updated guideline from the Joint British Diabetes Society for Inpatient Care. Diabetic medicine: J Br Diabetic Assoc. (2022) 39:e14788. doi: 10.1111/dme.14788 35224769

[44] Lo Preiato V Salvagni S Ricci C Ardizzoni A Pagotto U Pelusi C. Diabetes mellitus induced by immune checkpoint inhibitors: type 1 diabetes variant or new clinical entity? Review of the literature. Rev endocrine Metab Disord. (2021) 22:337–49. doi: 10.1007/s11154-020-09618-w 33409866

[45] Clotman K Janssens K Specenier P Weets I De Block C. Programmed cell death-1 inhibitor-induced type 1 diabetes mellitus. J Clin Endocrinol Metab. (2018) 103:3144–54. doi: 10.1210/jc.2018-00728 29955867

[46] Arima H Iwama S Inaba H Ariyasu H Makita N Otsuki M. Management of immune-related adverse events in endocrine organs induced by immune checkpoint inhibitors: clinical guidelines of the Japan Endocrine Society. Endocrine J. (2019) 66:581–6. doi: 10.1507/endocrj.EJ19-0163 31243183

[47] Schneider B Naidoo J Santomasso B Lacchetti C Adkins S Anadkat M. Management of immune-related adverse events in patients treated with immune checkpoint inhibitor therapy: ASCO guideline update. J Clin oncology: Off J Am Soc Clin Oncol. (2021) 39:4073–126. doi: 10.1200/JCO.21.01440 34724392

[48] Byun D Wolchok J Rosenberg L Girotra M. Cancer immunotherapy - immune checkpoint blockade and associated endocrinopathies. Nat Rev Endocrinol. (2017) 13:195–207. doi: 10.1038/nrendo.2016.205 28106152 PMC5629093

[49] Joshi M Whitelaw B Palomar M Wu Y Carroll P. Immune checkpoint inhibitor-related hypophysitis and endocrine dysfunction: clinical review. Clin Endocrinol. (2016) 85:331–9. doi: 10.1111/cen.2016.85.issue-3 26998595

[50] Elia G Ferrari S Galdiero M Ragusa F Paparo S Ruffilli I. New insight in endocrine-related adverse events associated to immune checkpoint blockade. Best Pract Res Clin Endocrinol Metab. (2020) 34:101370. doi: 10.1016/j.beem.2019.101370 31983543

[51] Brahmer J Abu-Sbeih H Ascierto P Brufsky J Cappelli L Cortazar F. Society for Immunotherapy of Cancer (SITC) clinical practice guideline on immune checkpoint inhibitor-related adverse events. J immunotherapy Cancer. (2021) 9. doi: 10.1136/jitc-2021-002435 PMC8237720 34172516

[52] Thompson J Schneider B Brahmer J Achufusi A Armand P Berkenstock M. Management of immunotherapy-related toxicities, version 1.2022, NCCN clinical practice guidelines in oncology. J Natl Compr Cancer Network: JNCCN. (2022) 20:387–405. doi: 10.6004/jnccn.2022.0020 35390769

[53] Percik R Larkin J Morganstein D. Endocrinopathies induced by immune checkpoint inhibitors: the need for clear endocrine diagnosis. Lancet Oncol. (2021) 22:905–7. doi: 10.1016/S1470-2045(21)00305-3 34197741

[54] Hua C Boussemart L Mateus C Routier E Boutros C Cazenave H. Association of vitiligo with tumor response in patients with metastatic melanoma treated with pembrolizumab. JAMA Dermatol. (2016) 152:45–51. doi: 10.1001/jamadermatol.2015.2707 26501224

[55] Freeman-Keller M Kim Y Cronin H Richards A Gibney G Weber J. Nivolumab in resected and unresectable metastatic melanoma: characteristics of immune-related adverse events and association with outcomes. Clin Cancer research: an Off J Am Assoc Cancer Res. (2016) 22:886–94. doi: 10.1158/1078-0432.CCR-15-1136 PMC4755809 26446948

